# Building Quality Control for Molecular Assays in the Global Measles and Rubella Laboratory Network

**DOI:** 10.3390/vaccines12080824

**Published:** 2024-07-23

**Authors:** Bettina Bankamp, Raydel Anderson, Lijuan Hao, Elena Lopareva, Min-hsin Chen, Gimin Kim, R. Suzanne Beard, Yoshio Mori, Noriyuki Otsuki, Akihido Ryo, Paul A. Rota

**Affiliations:** 1 Centers for Disease Control and Prevention, 1600 Clifton Road N.E., Atlanta, GA 30329, USA; rdo7@cdc.gov (R.A.); idn1@cdc.gov (L.H.); ecl1@cdc.gov (E.L.); zvp8@cdc.gov (M.-h.C.); ofi6@cdc.gov (G.K.); zho3@cdc.gov (R.S.B.); 2 Department of Virology III, National Institute of Infectious Diseases, Tokyo 208-0011, Japan; yoshiom@niid.go.jp (Y.M.); otsuki@niid.go.jp (N.O.); aryo@niid.go.jp (A.R.)

**Keywords:** external quality assessment, genotyping, measles, rubella

## Abstract

More than 100 laboratories in the World Health Organization Global Measles and Rubella Laboratory Network (GMRLN) perform nucleic acid-based methods for case confirmation of measles or rubella infections and/or strain surveillance (genotyping). The quality of laboratory data is critical to ensure that diagnostic results and country reports to regional verification committees are based on accurate data. A molecular External Quality Assurance (mEQA) program was initiated by the US-CDC in 2014 to evaluate the performance of laboratories in the network. The inclusion of testing for measles and rubella viruses, with a focus on detection and genotyping, plus the diversity of assays and platforms employed required a flexible and comprehensive proficiency testing program. A stepwise introduction of new evaluation criteria gradually increased the stringency of the proficiency testing program, while giving laboratories time to implement the required changes. The mEQA program plays an important role in many processes in the GMRLN, including informing plans for the training of laboratory staff, access to reagents, and the submission of sequence data to global databases. The EQA program for Local Public Health Institutes in Japan is described as an example for national mEQA programs. As more laboratories initiate molecular testing, the mEQA will need to continue to expand and to adapt to the changing landscape for molecular testing.

## 1. Introduction

The World Health Organization (WHO) Global Measles and Rubella Laboratory Network (GMRLN) comprises more than 700 laboratories that are classified as Sub-National Laboratories (SNLs), National Laboratories (NLs), Regional Reference Laboratories (RRLs), or Global Specialized Laboratories (GSLs) [1]. Almost all of these provide case confirmation for measles and rubella using serological methods, and at least 119 of the laboratories also perform nucleic acid-based molecular methods for case confirmation and/or strain surveillance (genotyping). The most commonly used methods for case confirmation are IgM enzyme immunoassays and real-time RT-PCR (rRT-PCR). The standard method for genotyping is the analysis of the 450 nucleotides at the end of the coding region for the nucleoprotein gene (N450) for the measles virus (MeV) [2] and of the 739 nucleotides in the E1 gene (E1-739) for the rubella virus (RuV) [3]; the sequence data are used to support the classification of cases as either imported or endemic, and are reported to the global sequence databases—MeaNS for MeV and RubeNS for RuV [4,5]. All six WHO regions have set elimination targets for measles and rubella and by 2022, 83 countries had a verified measles elimination status, while 98 countries had verified the elimination of rubella and congenital rubella syndrome [6,7].

The quality of laboratory data is critical to ensure that diagnostic results and genotyping information are based on accurate and reliable laboratory data. Countries are required to include the results of genotyping in the annual report to the Regional Verification Committee, underscoring the need for accurate sequence data. It is important to note that the GMRLN does not prescribe kits or methods for IgM detection, molecular detection, or genotyping; instead, laboratories have the flexibility to choose assays that can generate high-quality data. The GMRLN has implemented multiple steps and procedures to ensure a high quality of laboratory testing including confirmatory testing, distribution of a laboratory manual and protocols [8], an emphasis on quality during training, distribution of quality-controlled reagents through the International Reagent Resource (IRR [9]), accreditation visits, and the use of practice panels and External Quality Assurance (EQA) programs for serology and molecular tests. Many of these approaches are described in a companion publication [1]. This paper focuses on the molecular EQA (mEQA) program.

## 2. Molecular EQA Process

**Figure 1:**
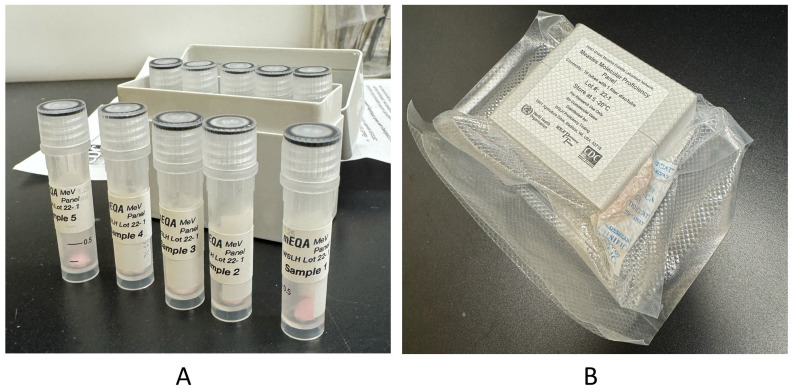
Measles and rubella proficiency panels consist of disks of FTA^®^ cards loaded with virus lysates. The figure shows a measles proficiency panel from the 2023 round of the mEQA program. (**A**) Tubes with FTA^®^ card disks. (**B**) Box sealed with desiccant to protect from humidity.

The introduction of a molecular proficiency testing scheme in the GMRLN required a solution to the problem of sample stability during transportation to international laboratories, especially those in countries with high ambient temperature. Flinders Technology Associates (FTA^®^) cards provide a means to ship samples as non-infectious materials at ambient temperatures [10]. Virus lysates dried onto FTA^®^ elute cards are rendered non-infectious by the chemicals the cards are impregnated with [10,11]. Panels consisting of tubes with 6 mm disks of virus-loaded FTA^®^ cards (Figure 1) provide a cost-effective approach to distributing proficiency samples that can be used for all steps of molecular testing, including RNA extraction.

Separate panels are prepared for MeV and RuV testing. Laboratories complete separate report forms for results of the MeV and RuV panels and submit them by email to the US-CDC, together with supplementary raw data such as rRT-PCR results (proprietary files from the platforms, exported data, or pictures of amplification curves), photos of gel electrophoresis, chromatograms, consensus sequences as fasta (text) files, etc. Due to the combination of detection and sequencing and the use of multiple different assays, there is a wide variety of supporting files that can be submitted. The report forms require participants to identify instrumentation, and specify methods, kits and reagents, expiration dates, and controls. This has resulted in a database that has proven useful in cases when laboratories encounter problems; because it allows the US-CDC to connect laboratories that use the same platform or reagents to share experiences. Feedback reports are returned from the US-CDC to the submitter with pass/fail evaluations. Regional laboratory coordinators (RLCs) and the global laboratory coordinator (GLC) are included in all steps of the process, e.g., the identification of laboratories that will participate in upcoming rounds of the mEQA program and the discussion of retest and remedial actions for laboratories with failing scores.

## 3. Changes over the Years

**Figure 2:**
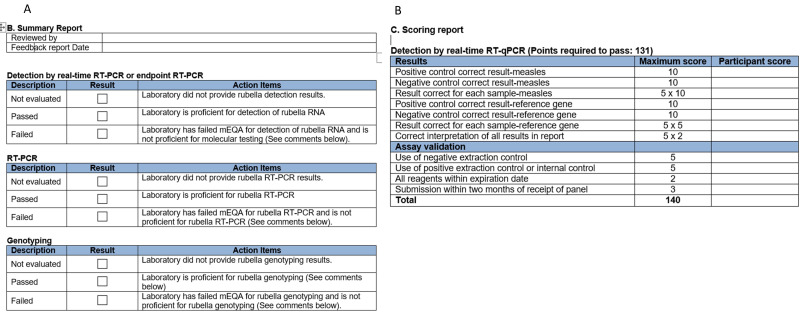
Excerpt from the feedback report. Since 2022, evaluation has been divided into three parts: detection, RT-PCR to amplify DNA for sequencing, and genotyping (sequencing and sequence analysis). Laboratories may participate in all or only some parts. Laboratories may pass or fail each part individually; (**A**). Summary report, (**B**). Scoring report.

The first lots of the panels were produced at the US-CDC but in 2016, production was moved to a vendor, with most quality control checks of the panels performed at the US-CDC. Initially, this vendor was responsible for shipping the panels but since 2020, shipping has been performed through the IRR [9], which also distributes kits and reagents for the GMRLN. In 2024, the IRR is expected to take over the production of the panels, which will centralize all production of kits and panels at one vendor. After the pilot round in 2014, the US-CDC provided panels for all WHO regions except the European Region, which had a separate mEQA scheme for MeV and RuV, albeit in close collaboration with the US-CDC to harmonize panels and evaluations. Since 2018, the US-CDC has provided panels for all WHO regions. This simplified planning for upcoming rounds of the mEQA program and ensured that laboratories from all WHO regions were evaluated based on the same criteria. Evaluation was divided into detection and genotyping in 2016, and genotyping was further subdivided into evaluation for RT-PCR and sequencing/sequence analysis in 2022. These divisions acknowledge that some laboratories do not perform all three parts of the mEQA program. Division into sections also allows laboratories to separate success in one part of the mEQA program from failure in another (Figure 2).

In 2019, the number of samples in each panel was increased from four to five. In the upcoming round for 2024, a sixth sample will be added to evaluate the ability of participating laboratories to detect samples with low viral load. In 2019, evaluation was changed from a qualitative pass/retest/fail system to a flexible numerical scoring system. This system facilitates the addition of new evaluation criteria and a gradual increase in the number of points awarded for newly added criteria.

## 4. Expansion and a Requirement for Flexibility

**Figure 3:**
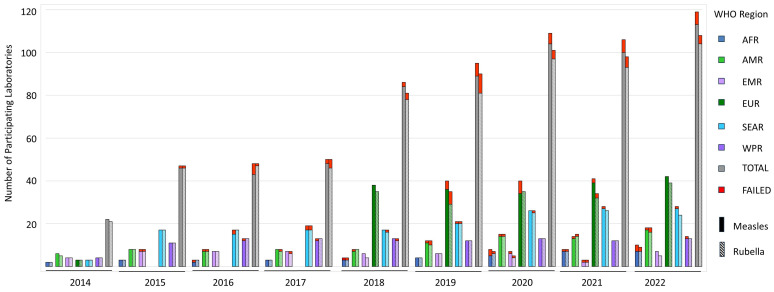
Number of participating laboratories per WHO region from 2014 to 2022. “Failed” depicts the number of laboratories that did not pass a retest. Note: The European Region did not participate in 2015–2017.

The pilot round of the mEQA program was conducted in 2014 with 22 participating laboratories. In the panel for 2022, 119 (MeV) and 108 (RuV) NLs and SNLs from all six WHO regions participated (Figure 3); a similar number is expected to complete the ongoing 2023 mEQA program round.

This expansion was fueled in part by the introduction of molecular methods into many NLs and SNLs during the COVID-19 pandemic. However, the equipment, testing needs, and level of experience vary widely among these laboratories. The African Region has made progress in the past 10 years in increasing the number of laboratories with the capacity to perform molecular assays (and consequently participate in the mEQA program), but is still not on par with other regions. The building of laboratory capacity in the Eastern Mediterranean region has been affected by armed conflicts in the region. The GMRLN is organizing training courses and using a “train the trainers” approach to increase capacity in these regions. In addition, the IRR provides low-income countries with reagents to ensure that measles surveillance is not affected by reagent stock-outs. The inclusion of testing for measles and rubella, a focus on detection and genotyping, plus the diversity of assays and platforms employed required a flexible and comprehensive proficiency testing program. A few laboratories participate only in the MeV or RuV portion of the mEQA program. Some laboratories participate only in the detection part, others in both detection and genotyping and/or sequencing and sequence analysis. Some laboratories perform their own sequencing, others transfer PCR products to sequencing laboratories but analyze the raw sequencing data. This diversity differentiates the MeV and RuV mEQA scheme from other proficiency testing schemes [12,13,14,15] and requires corresponding flexibility in the report forms and the evaluation of results; it also requires the evaluators to possess considerable expertise in multiple different assays.

## 5. Improving Quality through Increased Demands in the mEQA Program

The criteria for passing have been significantly strengthened over the years, reflecting the philosophy of the MeV and RuV mEQA program as a constantly evolving process that introduces continuous improvement in quality in participating laboratories. All proposed changes are discussed with RLCs and the GLC before their introduction. New criteria are introduced in a stepwise fashion, usually in the first year as non-scored comments, giving laboratories time to implement the required changes and preventing large numbers of laboratories from failing. In the following year, these new criteria are scored with a low number of points, allowing laboratories to still pass, but highlighting the importance of adapting to the increased demands. For example, a detection assay for a human reference gene is important to assess specimen quality and to distinguish between negative and invalid samples. Increased emphasis on inclusion, correct execution, and interpretation of a reference gene rRT-PCR over several years has reduced the number of laboratories that perform rRT-PCR detection assays without reference genes from 10 in 35 participating laboratories (2017) to 1 in 98 (2022), while keeping the annual number of failures low (Figure 3).

## 6. Remedial Procedures

If a laboratory receives a failing score, the RLC and, in case the failing laboratory is an RRL, the GLC is contacted to discuss options. An immediate retest can be offered in cases where the cause for failure is easily addressed, such as re-analyzing sequence data. In other cases, such as difficulties with interpreting rRT-PCR results, the laboratory will remain in a failed status until either remedial training or a site visit can be conducted. If the problem lies with data management issues (e.g., sending the wrong sequence file), a root cause analysis and corrective action plan will be requested from the laboratory. While a greater number of laboratories may fail initially, this number is reduced after retesting. For example, 14 of 105 laboratories failed the MeV mEQA program in the 2022 round, and 10 of 98 laboratories failed the RuV mEQA program; however, after successful retesting, only 6 and 4 laboratories, respectively, remained in the failed status. Retesting allows laboratories to learn from and correct errors immediately without causing longer-term interruptions.

Over the years, the mEQA program has acquired a central role in many processes in the GMRLN. The results identify training needs in individual laboratories, while trends in inadequate results can inform training activities for entire WHO regions. The successful completion of the mEQA program is required to obtain molecular kits and reagents from the IRR (except for the panels and a small number of kits and reagents to practice the assays). Laboratories that fail the genotyping part of the mEQA program are not allowed to submit sequences to MeaNS or RubeNS, to prevent the submission of potentially incorrect sequences. To prevent interruption in molecular surveillance, these laboratories are required to develop a contingency plan with their RLCs, which may include sending specimens to another laboratory for sequencing and sequence analysis.

## 7. Sub-National mEQA Programs

An increasing number of countries are building sub-national laboratory networks. SNLs decentralize testing, reduce shipping distances, and increase surge capacity to test large numbers of specimens during outbreaks. However, the mEQA program administered by the US-CDC does not have the capacity to provide proficiency panels to SNLs. Instead, countries with large or growing networks of SNLs are encouraged to develop a national mEQA program. One example is the national mEQA program in Japan, where Local Public Health Institutes (LPHIs) have been established by prefectures or government-designated cities as public testing laboratories that contribute to local public health. LPHIs conduct tests for various infectious diseases as defined in the Infectious Diseases Law, and report to the National Institute of Infectious Diseases (NIID) when pathogens are detected.

The Japanese Ministry of Health, Labour and Welfare (MHLW) established guidelines for measles and rubella control in 2007 and 2014, respectively, with the target of eliminating these diseases. Under the guidelines, LPHIs are required to conduct molecular detection for all measles cases starting in 2013 and for RuV cases starting in 2018. Furthermore, if measles or RuV viruses are detected in the tests, LPHLs or the NIID are required to conduct nucleotide sequencing and genotyping.

Since the fiscal year 2016, the MHLW has launched an external quality assessment program for pathogen testing and has commissioned the NIID to conduct the program. Participation is recommended for LPHIs that do not participate in other proficiency testing programs. Two to three pathogen tests are set as assignments each year, from which LPHIs select at least one. For MeV and RuV, molecular detection was tested in the fiscal years 2018 and 2023, and genotyping was evaluated in the fiscal year 2019. Between 68 and 79 LPHIs, which were the majority of laboratories that were conducting measles–rubella testing at that time, participated in these assignments. For assignments in molecular detection, participating laboratories were asked to test multiple blinded samples containing MeV and RuV viruses using the methods that were usually performed. For the genotyping assignment, samples already known to be positive for MeV and RuV were provided for nucleotide sequencing, phylogenetic tree analysis, genotyping, and the naming of viral strains. In these assignments, most LPHIs submitted correct results, but a small number of LPHIs answered incorrectly or had problems (e.g., setting controls). The purpose of this program is not to evaluate or certify the eligibility of laboratories, but to provide an opportunity for each laboratory to check its own testing proficiency. However, when the NIID identifies a false positive or a problem, it points out the problem, provides technical advice and training, and assists the laboratory in solving the problem.

## 8. Considerations for the Development of National EQA Programs

While every country may face unique challenges, there are general considerations that can support the development of a national EQA program, as follows:

1. Identification of laboratories to participate in initial rounds. In some countries participation may be voluntary, while in others, laboratories may be selected by the government.
2. Defining the scope of testing. Countries that are not yet close to the elimination of measles and/or rubella may focus on serological methods for outbreak confirmation and may not need molecular diagnostic assays but should instead focus on strain surveillance by SNLs. On the other hand, some countries may have SNLs that perform molecular diagnostic assays but send positive specimens to the NL for sequencing, obviating the need for an EQA program for strain surveillance. It is also possible to separate the detection and strain surveillance parts into two separate programs.
3. Identification and testing of sample distribution method. Countries with the resources for the fast shipping of frozen samples may choose to generate panels from pooled clinical specimens, which have the advantage of being most like the specimens the laboratories will receive. Countries in which the shipping of frozen samples is logistically challenging may prefer more stable sample types such as FTA^®^ cards. Sample options include cell culture-grown virus stock and extracted RNA. Regardless of sample type, the NL should test the stability of the samples under conditions expected in the field (e.g., elevated ambient temperature).
4. Development of a reporting structure. This includes both the reporting mechanism (email or dedicated website with uploading function) and reporting content. For example, what results should be reported? The mEQA program in the GMLRN created a report form that includes questions about the equipment and reagents used. The report form also explains which additional documents should be submitted (e.g., phylogenetic tree).
5. Execution of a pilot round with a small number of high-performing laboratories. The submitted results should not be scored. Instead, the NL should give feedback to the participants to improve their understanding of expectations and encourage feedback from the participants.
6. Development of a scoring system. The mEQA program in the GMRLN started with a simple pass/fail system but later moved to a numerical scoring system. Stringency should be increased over time to avoid large numbers of participants failing the EQA program.
7. Definition of remedial actions for under-performing laboratories. Will there be an option for a repeat? Will laboratories that do not pass be allowed to continue testing? If yes, what measures will be taken to ensure high-quality results? If no, what steps will be taken to ensure continued molecular surveillance?

## 9. Challenges and Future Developments

Compared to clinical specimens, the RNA copy number in proficiency panels is in the normal-to-high range in the MeV proficiency panels and very high in the RuV panel. Therefore, the panels do not represent the range of viral loads in clinical samples. The copy number was chosen to ensure that the samples give positive signals in detection assays despite inconsistent storage conditions while in transport and can be successfully sequenced. With shipping, the time for testing and possible retesting, and the evaluation of results, a round of the mEQA program takes almost a full year. Over the course of the project, we found that while virus lysates on FTA^®^ cards are more stable than other forms of distribution (e.g., dried RNA), they are not stable at ambient temperature for extended periods of time, especially in tropical countries. However, the high RNA copy number does not allow for an assessment of the sensitivity of detection of the assays used in the participating laboratories. This issue will be addressed in the 2024 round of the mEQA program with the addition of unscored samples with lower RNA copy numbers for MeV and RuV.

Another limitation is that the turnaround time for reporting mEQA program results is currently three months, while laboratories are expected to submit sequence data from current cases to MeaNS/RubeNS within two months of receipt of the specimen. Three months was chosen as the time limit for proficiency testing to give laboratories ample time to complete the genotyping part of the proficiency testing. However, if molecular assays are used to confirm cases, results should be submitted to the national program within four days. RLCs have suggested separate reports for the detection and the sequencing parts of the mEQA program, and this change in reporting will be introduced in the 2025 round of the mEQA program.

A global EQA program faces practical challenges such as stability of the panel during shipping; shipping delays; language issues complicating the use of the report form; staff turnover in participating laboratories affecting access to the IRR for ordering the panels and reagents; laboratories experiencing difficulties in procuring equipment maintenance; and even the possibility of laboratories sharing results. Managers of the mEQA program at the US-CDC Atlanta maintain communications with RLCs to identify and address these challenges, for example, through ongoing stability testing of the panels under elevated temperature conditions for each round of the mEQA program until the testing has been completed in all laboratories; deadlines of three months after receipt of the panel to allow laboratories to address reagent shortages or equipment maintenance problems; communication between evaluators and submitting laboratories if ancillary documents with raw data are missing or questions arise over data entered into the report form; and creation of multiple versions of the panel.

The reduction in circulating MeV genotypes has made routine genotyping data less informative [16] and has created the need to sequence larger regions of the MeV genome, either the non-coding region between the matrix gene and fusion gene coding regions (MF-NCR) or the whole genome [17,18,19,20]. Whole genome sequences (WGSs) of RuV are also generated, especially for the analysis of persistent infections [21,22,23]. Sanger sequencing is used for genotyping in most of the GMRLN laboratories and is also the standard method for the sequencing of the MF-NCR. It is expected that laboratories will transfer the stringent quality control criteria used for routine genotyping to Sanger sequencing of the MF-NCR region. WGSs are usually produced using Illumina^®^ or Oxford Nanopore Technologies^®^ platforms. A separate EQA program for these sequencing methods would ensure that the GMRLN laboratories are proficient in the use of these new sequencing platforms and that MeV MF-NCR sequences and MeV or RuV WGSs submitted to MeaNS and RubeNS are of high quality.

As stated above, there are no prescribed assays. This flexibility allows laboratories to choose the most appropriate assay, but it complicates the administration of the mEQA program. For example, the introduction of testing for a reference gene was delayed by some laboratories that were reluctant to replace nested end-point RT-PCR assays, which cannot easily accommodate testing for a reference. The different approaches to sequencing (in-house, in a GMRLN RRL, by a commercial vendor) continue to complicate the evaluation of results. The COVID-19 pandemic has initiated a move away from Sanger sequencing towards newer platforms, requiring an adaptation of the evaluation and scoring system.

## 10. Conclusions and Perspective

The success and expansion of the GMRLN is based on the collaboration between colleagues from different regions of the world who all share the goal of improving laboratory testing in support of their countries’ measles and rubella elimination goals. The purpose of the MeV and RuV mEQA program is to build capacity for high-quality testing in the network. The mEQA program has proven to be an essential tool for the evaluation of the quality of molecular tests in network laboratories and has acquired a central role in many processes in the GMRLN. The stepwise introduction of increased requirements for passing has improved the quality of molecular testing without putting unnecessary pressure on the laboratories. The mEQA program will need to continue to adapt to the changing landscape in molecular testing, especially considering the rapid evolution of sequencing methods. With the continued expansion of molecular testing, particularly in the African Region, the number of participants in the MeV and RuV mEQA program and the number of countries with independent, sub-national EQA programs are expected to grow.

## Data Availability

No new data were created.
